# Spatial navigation ability is associated with the assessment of smoothness of driving during changing lanes in older drivers

**DOI:** 10.1186/s40101-020-00227-9

**Published:** 2020-08-27

**Authors:** Masafumi Kunishige, Hideki Miyaguchi, Hiroshi Fukuda, Tadayuki Iida, Kawabata Nami, Chinami Ishizuki

**Affiliations:** 1 Division of Occupational Therapy, Graduate School of Biomedical & Health Sciences, Hiroshima University, 1-2-3 Minamiku Kasumi, Hiroshima City, Hiroshima Pref 734-8551 Japan; 2 Department of Human Behavior Science of Occupational Therapy, Health Sciences Major, Graduate School of Biomedical & Health Sciences, Hiroshima University, 1-2-3 Minamiku Kasumi, Hiroshima City, Hiroshima Pref 734-8551 Japan; 3 Graduate School of Information Sciences, Hiroshima City University, 3-4-1 Ozukahigashi Asaminami-ku, Hiroshima City, Hiroshima Pref 731-3166 Japan; 4 Department of Physical Therapy, Faculty of Health and Welfare, Prefectural University of Hiroshima, 1-1 Gakuencho, Mihara City, Hiroshima Pref 723-0053 Japan; 5 Department of Rehabilitation/Occupational Therapist, Faculty of Health Sciences, Hiroshima Cosmopolitan University, 3-2-1 Ozukahigashi Asaminami-ku, Hiroshima City, Hiroshima Pref 731-3166 Japan

**Keywords:** Driving, Older drivers, Driving simulator, Spatial navigation, Driving ability

## Abstract

**Background:**

Age-related changes affect driving ability, including the smoothness of driving. This operation requires the use of both allocentric strategies (based on world-centered representations) and egocentric strategies (based on self-centered representations); however, with age, a greater preference for egocentric strategies is evident when driving. Furthermore, an age-related decline occurs in both driving ability and spatial navigation. We therefore assessed the relationship between spatial navigation and driving smoothness and tested whether a driving simulator can be used to evaluate smooth lane changes in older drivers.

**Methods:**

A total of 34 healthy older drivers (mean age: 68.2 ± 5.4 years old) and 20 younger drivers (mean age = 20.2 ± 5.4 years old) participated in this study. The smoothness of driving was assessed using a driving simulator and spatial navigation was assessed using the Card-Placing Test-A/B. We also assessed visual perception and general intellectual function using standard neuropsychological tests.

**Results:**

Older drivers had significantly worse spatial navigation and exhibited less smooth driving than younger drivers. Furthermore, we found a negative correlation between the smoothness of driving and spatial navigation within both groups. These results suggest that the deterioration in spatial navigation in older people may underlie the observed decrease in driving smoothness, and that spatial navigation and smooth driving deteriorate with age.

**Conclusions:**

Considering these results, we found a significant correlation in the older group between the smoothness of vehicle movement and spatial navigation, in the smoothness of vehicle movement between the young and old groups. The smoothness values, which indices thoroughly derived from the driving simulator are indeed showing some evidence in ego/allocentric cognitions, which may change by age. The driving simulator could aid the development of intervention programs or assessment measures for drivers with a decreased function.

## Background

Spatial navigation ability is essential for everyday living, allowing us to be cognizant of our position and orientation in our environment. This ability consists of several components, spatial memory, sensorimotor information, sense of direction, and spatial reference frames (the egocentric frame and the allocentric frame). These components interact with each other in various situations. Spatial navigation is the process that determines the trajectory from one place to another [1]. Successful spatial navigation required translation of encoded survey-level map information for orientation and implementation of a planned route to the goal and mainly relies on two co-dependent strategies: allocentric and egocentric navigation strategies. These strategies use different types of spatial reference frames but are highly correlated [2]. The deficit of this process increased safety concerns, greater risk of getting lost, and reduced driving confidence for adults [3]. The egocentric frame includes spatial information about the location of oneself in the environment, and the allocentric frame involves spatial information about the position of objects relative to each other. The egocentric frame is based on subject-to-object relations and leads to body-centered representations (self-centered representations). The allocentric frame is acquired later in life [4]; within this reference, locations are described using object-to-object relationships, independently from the subject’s point of view (world-centered representations). While males typically outperform females in tests of spatial navigation, significant female advantages have been documented in spatial memory [5]. One study pointed out almost all the previous spatial impairments, both regarding egocentric preference and allocentric/switching deficits, considering both navigation and spatial memory [6]. In other words, in the process of spatial navigation, a driver who can switch egocentric and allocentric frames with a good balance operate the car smoothly. To the contrary, the reason why driving is awkward is the balance between egocentric and allocentric frames due to aging is biased. Moreover, it is thought that there is a problem that it is difficult to switch between spatial navigation components using the frontal lobe responsible for the executive function in even normal people. These problems can cause the driver to get in the wrong direction, delay the time to change lanes, and not drive smoothly.

Two experimental studies have indicated there to be an age-related preference for using egocentric rather than allocentric strategies during navigation [7, 8]. Declines in spatial navigation ability are reportedly caused by frontal lobe atrophy, such as white matter abnormalities in the prefrontal and frontal cortices, atrophy of frontal gray matter [9, 10], and striatal volume reduction [11]. With aging, the reduced capacity for allocentric spatial information processing may be due to frontal lobe atrophy [12]. Driving is a complex multitasking activity that involves perception, attention, decision-making, sensory, motor, and higher-level cognitive components and spatial navigation. Spatial navigation may be particularly relevant for understanding changes in driving.

Many studies have interpreted this change in driving ability as a decline in attention, as indicated by findings of a correlation between the Trail Making Test scores and accident history [13, 14]. Furthermore, older people report substantial declines in navigational capabilities [15], which can severely restrict mobility. Indeed, an age-related decline in spatial navigation ability has been reported [16]. In other words, we expected to see a maintenance in selective attention to visual information (such as the direction of movement and signs) necessary for driving to decrease with increasing age, even though driving operation ability somehow remains. When changing lanes, the driver estimates the distance from other vehicles and plans a route to change lanes. After that, a driver will typically check the speedometer, the side mirror, and the driving direction, will judge the speed of one’s own vehicle and the positional relationship with other vehicles, and then change lanes. Therefore, the egocentric frame in the cognitive process retrieves visual information to visually check equipment, such as the speedometer and the driving direction, and planning the route by sensing the distance and positional relationship with other vehicles. It seems that the allocentric frame is functioning in a cognitive process that monitors change of vehicle trajectory and the surrounding environment from a bird’s-eye view. As the function of allocentric declines, there is a possibility that the vehicle operation cannot be performed smoothly.

The driving simulator(DS)can be used to measure driving ability in a realistic, safe, and controlled driving environment. In particular, it has been shown that speed fluctuation can be important factors for assessing driving technique in lane changing [17, 18]. From the viewpoint of smooth operation, there are researches on lane change path generation using the lateral acceleration and jerk of the vehicle when changing lanes as an evaluation function [19]. From these previous studies, we considered that a DS can be used to evaluate whether lane change was performed smoothly. In our previous study, it has reported that eye movement is increased in older people with reduced spatial navigation ability when changing lanes on DS [20]. In particular, using the right turn and lane change at the intersection, the attention function (more eye movements) is required at the right turn, and the space navigation ability is required at the lane change. In this study, we chose to change lanes as a task requiring spatial navigation ability without using the attention function. Spatial navigation at the time of driving does not depend on one strategy alone, but the ability to flexibly switch between various space strategies according to the surrounding environment and combine these as necessary. Since DS is a two-dimensional task, it does not capture things in three dimensions. There is a certain validity and we think that DS can measure 3D space task.

The purpose of this study was to test the hypothesis that some drivers whose spatial navigation ability has deteriorated are less able to perform smooth lane change. We selected a simple task—lane changing—to assess smoothness during driving operation. The first derivative of the position in the lateral direction (*y* direction) with respect to the position in the driving direction (*x* direction) of the vehicle body, and the root mean square (RMS) of the *x* direction were used as the evaluation index of smoothness. Spatial navigation ability was assessed using the neuropsychological test. Our findings indicate that DS can be used to evaluate driving ability in older adults, which has important practical implications for driving safety.

## Methods

### Study design and participants

The participants were healthy older and young adults living in Hiroshima Prefecture, who were voluntarily recruited through public recruitment to the present study. Initially, a total of 43 people responded; 23 young people were initially interested. We explained the study details to participants in advance and collected data from those participants who provided informed consent. The inclusion criteria were (1) without eye diseases, (2) intact physical function, and limb movement ability. The exclusion criteria were as follows:

①Having a mental illness such as depression

②With a diagnosis of dementia

③Difficulty in communication and cannot answer the questions during the interview

④ With history with motion disease and restriction, such as heart disease and disorder of brain function), and there is a danger of sudden change or deterioration in health condition during the study

⑤Difficulty in the following measurement during the study.

**Fig. 1:**
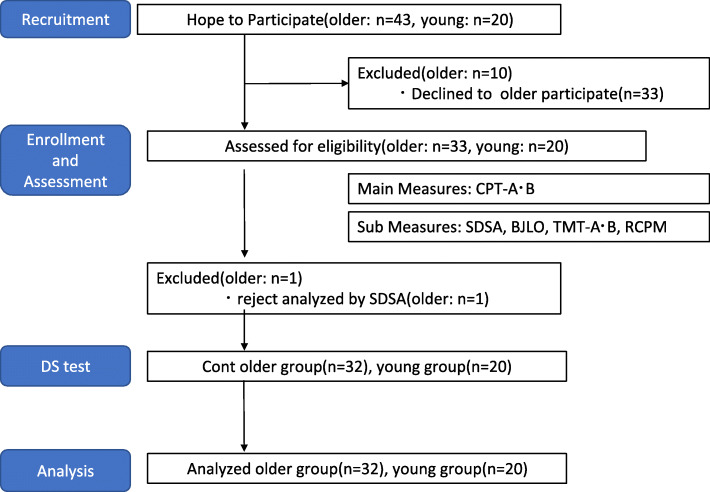
Flow Diagram of Conte group

After application of these criteria, eligible participants included 34 healthy older Japanese people (female: *n* = 27; male: *n* = 7; age: (mean and standard deviation [SD]) 68.2 ± 5.4 years old; range: 60 to 76 years old) and 20 young people (female: *n* = 10; male: *n* = 10; age: 20.2 ± 5.4 years old; range: 22 to 30 years old). The older group had held a driver’s license for a mean of 38 years and had a mean driving mileage per year: 7740 ± 721.1 km; the younger group had held a driver’s license for a mean of 5 years and had a mean driving mileage per year: 8240 ± 935.2 km. We explained the study details to participants in advance and informed consent was provided by all participants. This study was approved by the Epidemiological Research Ethics Review Committee of Hiroshima University (approval number E-1003) and was conducted in compliance with the Declaration of Helsinki. We defined DS experienced persons in this study as those who had received a period of preliminary operation experience in a motion-based DS. Practice time (15 min) was set sufficiently for all participants. We needed to reduce the bias related to the driving operation in DS, and we used the following primary outcomes and secondary outcomes (Fig. 1).

### Outcome measures

#### Primary outcome: spatial navigation (Card-Placing Test-A/B)

The CPT-A/B was used to evaluate spatial navigation. In part A of the CPT, a subject must stand in the center square of nine squares arranged in three rows of three. The subject was instructed to remember the spatial locations of three different cards randomly placed in one of the eight squares. After 10 s, the cards were taken away and the subject is to restore them to their original positions. Part A of the test assesses the ability of a subject to retain information on spatial locations of cards placed on the floor around her/him (an egocentric reference frame, i.e., self-centered). Meanwhile, in part B, immediately after the cards had been removed, the subject was rotated to the right or to the left by 90° or 180° and then asked to replace the cards. The CPT-B requires the integration of the self-centered spatial representation and information about self-body rotation. In other words, CPT-B is considered to evaluate the spatial representation ability when information about the direction of the self-body with respect to the card position is required (allocentric reference frame), that is, the environment-centered spatial representation ability [21]. For both part A and part B of the CPT, the subject underwent 10 consecutive trials and scored 1 point if the location of a card that the subject replaced was correct. The full score of each of part A and part B of the CPT was 30 points.

### Secondary outcomes

#### Driving assessment (the Stroke Drivers’ Screening Assessment Japanese Version)

We used the Stroke Drivers’ Screening Assessment Japanese Version (SDSA) to evaluate whether the participant understand driving rules. The SDSA was developed as a screening assessment for drivers post-stroke and contains four tests, as follows: (1) the Dot Cancellation Test, (2) Square Matrices Directions, (3) Square Matrices Compass, and (4) the Road Sign Recognition Test. The SDSA has been reported to be relatively successful in predicting pass/fail classification of an on-road evaluation (78.9% agreement with the principal evaluator [22]; sensitivity, 71.4%–79.3%; and specificity, 77.8%).

#### Evaluation of visual perception (the Benton Judgment of Line Orientation Test)

Driving on the DS is displayed on a liquid crystal display. The Benton Judgment of Line Orientation (BJLO) test was used to evaluate visual perception. The BJLO tests whether the direction in 2D space can be recognized [23]. This test includes 30 pairs of angled lines to be matched with the corresponding lines in a radially arranged 11-line display. The full score of BJLO was 30 points.

#### Screening test of general intellectual function (Raven’s Colored Progressive Matrices)

We used Raven’s Colored Progressive Matrices (RCPM) to ensure that the ability to follow non-verbal instructions during driving, such as signs and signals, was preserved. This test requires participants to judge the relationship between identity and similarity of, and differences between, graphics. Participants must choose one out of six figures that seems to fit a part of the displayed designs and designs that incomplete figure. Because RCPM scores have been found to show a strong correlation with those of the Wechsler Adult Intelligence Scale-Revised (WAIS-R), it is also known as a screening for the simple intelligent test visual perception in the medical field [24]. The full score of RCPM was 36 points.

#### Evaluation of visual search (Trail Making Test A and B)

We assessed driving concentration during DS implementation using the Trail Making Test A and B (TMT-A and TMT-B), which measure processing speed [25]. Part A is administered as a baseline measure of motor and visual search speed, whereas part B is administered as a measure of set-shifting and inhibition. The TMT-B simply adds the order of letters (e.g., a–b–c …) to the numbers by connecting lines in order (e.g. 1–2–3 …) and adding numbers and letters, and the task is to connect lines alternately on paper. The participant should complete the task while changing the two elements [26]. The time required to switch attention gradually decreases with age, and if the time required to complete the TMT-B is 180 s or more, there is no cause for dementia or depression, it is necessary to confirm [27]. We measured the time spent on the assignment.

#### Experimental driving simulator device

**Fig. 2:**
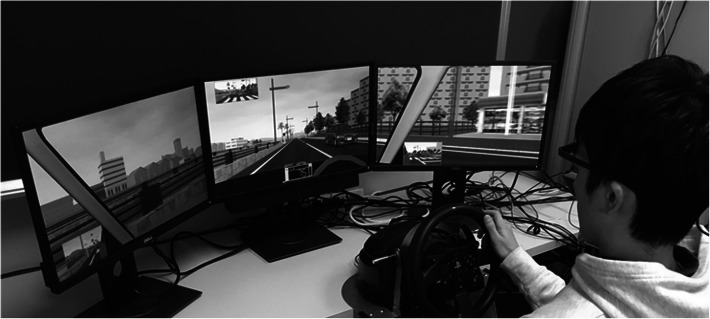
Experimental layout. The DS used in this study was a modified version of the Honda Safety Navi system, right-hand-drive version (Honda Motor Co., Tokyo, Japan), a DS used for instruction in efficient, safe driving

For the DS, which has been previously described in detail [20], we used a revised version of the Honda Safety Navi system (Honda Motor Co, Tokyo, Japan) (right-handed drive version) (Fig. 2). DS used in this study was a modified version, which is a DS used for instruction in efficient, safe driving. Information such as steering and pedal operation, vehicle body coordinates, and speed, as well as events such as the scenario type, were stored as logs at sampling intervals of 10 ms. When using part of the course of the scenario provided by the main software for the experiment, it is necessary to keep the log even if it is forcibly terminated in the middle of the scenario. Therefore, we used User Datagram Protocol communication to transmit the coordinates, speed, steering angle, accelerator pedal, brake pedal depression amount, turn signal status, and signal state.

#### Experimental DS procedure

**Fig. 3:**
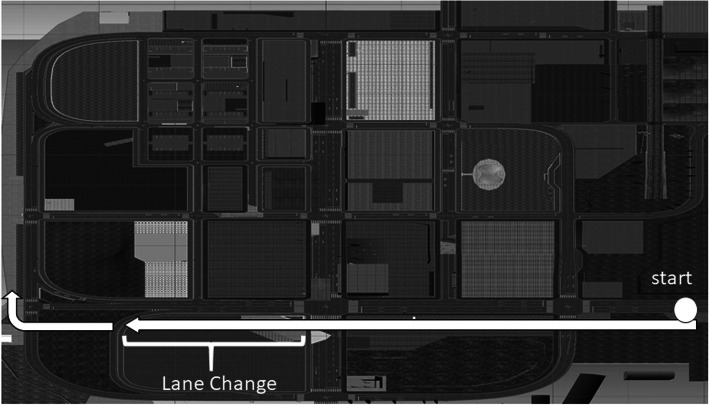
Route of driving and snapshot of a driving movie. This scene is the screenshot before changing the lane

We tested using the DS after performing neuropsychological examinations. We used an experimental course that simulated an urban area, as shown in Fig. 3. The course environment was set to be equivalent to a dry road surface in the daytime, and the friction on the road surface was set to *μ* = 0.75. In this urban district risk prediction course, dangerous event scenarios included oncoming cars and children jumping out in front of the car. Immediately before the main test, participants completed a risk prediction practice course. The scenario in this practice course was different from the scenario used in the actual experiment. Participants completed a 15-min practice that included one round of the course. Participants subsequently attempted the set course five times (5 × 2-min main tests). Participants were asked to drive in compliance with the Road Traffic Act, follow the voice guidance while driving, and keep to the outer lane while turning at an intersection.

#### Data analysis

Between-group analysis of driving ability and neuropsychological results. The SDSA was used to screen drivers for abilities such as a sense of direction and an awareness of traffic rules. The SDSA scores determined whether participants should attempt the driving task. The score was substituted into the prediction formula, and the numerical value was calculated. Two-sample *t* tests were used for between-group comparisons on the BJLO, CPT (A and B), TMT (A and B), and RCPM scores.

**Fig. 4:**
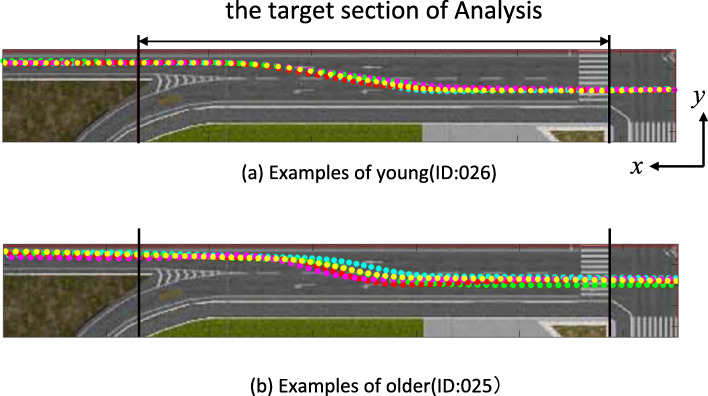
Route of driving. In this study, we measured the trajectory during a lane change. We analyzed driving data from intersection (*x* = 0) to after lane change (*x* = 105)

**Fig. 5:**
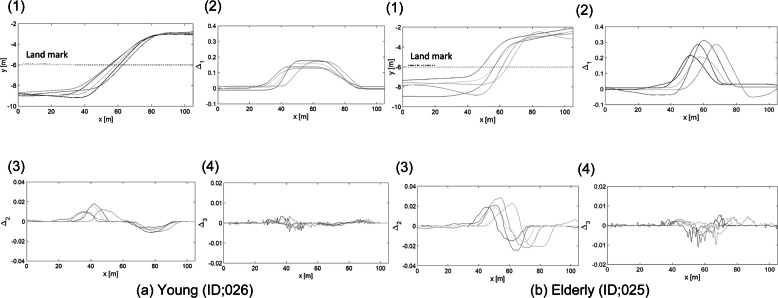
Measurement data example of the target section. We analyzed driving data from intersection to after lane change. There are the pass of example drivers. **a** Examples of young (ID: 026) and **b** examples of older (ID: 025). The example of data obtained from DS during driving. For example **a** young and **b** older. (1) The *X*, *Y* coordinates were calculated from the sampled data, and the car body angle and the steering angle at each point were calculated. (2) Δ1:RMS of the first derivative of the position in the lateral direction with respect to the position in the driving direction, (3)Δ2:RMS of the second derivative of the position in the lateral direction with respect to the position in the driving direction, (4)Δ3: RMS of the third derivative of the position in the lateral direction with respect to the position in the driving direction

To evaluate the smoothness of lane change, the first derivative, second derivative, and third derivative of the position in the lateral movement (*y* direction, as shown in Fig. 4) relative to the position in the driving direction (*x* direction, as shown in Fig. 4) of the RMS was used as an evaluation index (Fig. 5). Because there was no limit in the speed of driving direction at the time of lane change, the number of data samples in the analysis section in the driving direction differed between participants. All participants were asked to operate the DS under the same conditions according to the voice instructions. The same start position is set, and the data are analyzed to calculate the values at each operation. Therefore, the data samples were aligned at 10 cm intervals in the *x* direction, and the position in the *y* direction was calculated using cubic spline interpolation. Assuming that the position data of the *i* sample after interpolation is (*x* (i), *y* (i)), the evaluation index was calculated using RMS∆1. However, if *x* (0) and *x* (N-1) were the *x*-coordinate data of the start and end of the target section, the three samples (*x* (− 1), *x* (− 2), *x* (− 3)) were also used. (1) The *X*, *Y* coordinates were calculated from the sampled data, and the car body angle and the steering angle at each point were calculated. (2) Δ1:RMS of the first derivative of the position in the lateral direction with respect to the position in the driving direction, (3) Δ2:RMS of the second derivative of the position in the lateral direction with respect to the position in the driving direction, (4) Δ3: RMS of the third derivative of the position in the lateral direction with respect to the position in the driving direction.

The evaluation function RMS of the first derivative of the position in the lateral direction with respect to the position in the driving direction is defined as RMS∆1 (1).

1 $$ \mathrm{RMS}{\Delta }_1=\sqrt{\frac{1}{N}\sum \limits_{i=0}^{N-1}{\left\{{\Delta }_1(i)\right\}}^2} $$

$$ {\Delta }_1(i)=\frac{y(i)-y\left(i-1\right)}{x(i)-x\left(i-1\right)},i=-2,-1,0,1,\dots, N-1 $$

In the above equation, *N* represents the number of data samples after interpolation in the target section.

The evaluation function RMS of the second derivative of the position in the lateral direction with respect to the position in the driving direction was defined as RMS ∆2 (2)

2 $$ \mathrm{RMS}{\Delta }_2=\sqrt{\frac{1}{N}\sum \limits_{i=0}^{N-1}{\left\{{\Delta }_2(i)\right\}}^2} $$

$$ {\Delta }_2(i)=\frac{\Delta _1(i)-{\Delta }_1\left(i-1\right)}{x(i)-x\left(i-1\right)},i=-1,0,1\dots, N-1 $$

The evaluation function RMS of the third differentiation of the position in the lateral direction with respect to the position in the driving direction was defined as RMS ∆3 (3)

3 $$ \mathrm{RMS}{\Delta }_3=\sqrt{\frac{1}{N}\sum \limits_{i=0}^{N-1}{\left\{{\Delta }_3(i)\right\}}^2} $$

$$ {\Delta }_3(i)=\frac{\Delta _2(i)-{\Delta }_2\left(i-1\right)}{x(i)-x\left(i-1\right)},i=0,1,\dots, N-1 $$

We used Pearson’s correlation coefficient to calculate the association between BJLO, CPT (A and B), and RCPM scores, and RMS∆1, RMS∆2, and RMS∆3 when changing lanes.

## Results

### Between-groups comparison of neuropsychological results

Table 1 shows the mean scores for each test item in SDSA. Thus, one subject was excluded from the analysis subject. Neither group exhibited a pathologic decline as measured by the neuropsychological examination. Table 2 shows the mean test scores of the CPT-A/B, BJLO, TMT-A/B, and RCPM tests in older and younger participants. The young group performed significantly better than the older group on the CPT-B (*t* = 2.3, *p* < .001) and TMT-B (*t* = 4.5, *p* < .05).

**Table 1:** Comparison between SDSA Scores of older and young

		Older (*n* = 32)			Young(*n* = 20)		
		Mean	SD	Range	Mean	DS	Range
Dot Cancellation Test	Time(s)	544.12	175.53	288~864	434.33	73.32	173~598
	Errors	17.9	11.32	1~39	14.9	8.2	0~10
	FALSE	0.9	1.16	0~4	0.9	1.16	0~5
Square	Directions	25.63	4.45	16~32	29.51	2.15	24~32
Matrices	Compass	20.75	6.28	12~32	25.5	4.81	21~32
Road Sign Recognition Test		8.03	3.03	2~12	9.2	2.32	7~12

Comparisons of the distributed value of RMSΔ3 in lane change. The older group took significantly larger of RMSΔ3 in lane change

**Table 2:** Comparison between test scores (mean ± SD) of older and young (CPT-A/B, BJLO, TMT-A/B, and RCPM)

Test	Older	Range	Young	Range	*p*
CPT-A	27.01 ± 3.01	25~30	28.05 ± 1.91	27~30	n.s
CPT-B	20.36 ± 4.92	19~27	27.30 ± 2.10	25~30	*p* < .001
BJLO	26.82 ± 3.1	23~30	28.20 ± 1.76	26~30	n.s
TMT-A	23.96 ± 10.35	20~59	21.39 ± 4.98	17~30	n.s
TMT-B	57.34 ± 46.73	49~120	42.90 ± 9.98	28~58	*p* < .05
RCPM	34.67 ± 1.10	30~36	35.00 ± 1.35	33~36	n.s

Comparisons of the distributed value of RMSΔ3 in lane change. The older group took significantly larger of RMSΔ3 in lane change; mean ± SD

*n.s* not significant, *CPT-A/B* the Card-Placing Test Part A and Part B, *BJLO* Benton Judgment of Line Orientation Test, *TMT-A/B* the Trail Making Test Part A and Part B, *RCPM* Raven’s Colored Progressive Matrices

### Between-groups comparison of driving data

**Fig. 6:**
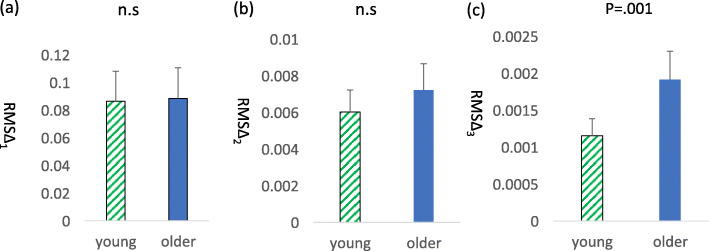
Comparisons of young and older **a** RMSΔ_1,_ **b** RMSΔ_2_, and **c** RMSΔ_3_ in lane change. The older group took significantly larger of the RMSΔ_3_ in lane change. n.s.; not significance

**Fig. 7:**
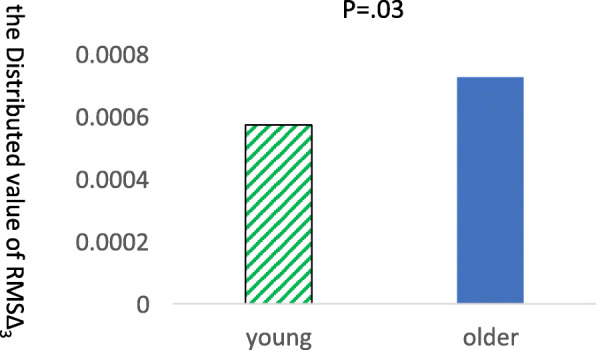
Comparisons of the distributed value of RMSΔ3 in lane change. The older group took significantly larger of RMSΔ3 in lane change

There was no significant between-group difference in average driving speed (average speed range: older group: 16.8 to 49.1 km/h; younger group: 19.5 to 54.3 km/h). Concerning between-group differences in the smoothness of driving, we found significant differences in RMS ∆ 3 (*t* = 0.56, *p* = 0.03) (Fig. 6) and the variance value of RMS ∆ 3 (*t* = 0.24, *p* = 0.01) (Fig. 7), whereby the variance value of RMS ∆ 3 was lower in the young group compared with the older group. There were no significant between-group differences in RMS ∆ 1 or RMS ∆ 2 (Fig. 6).

### Correlation between neuropsychological and driving results

**Fig. 8:**
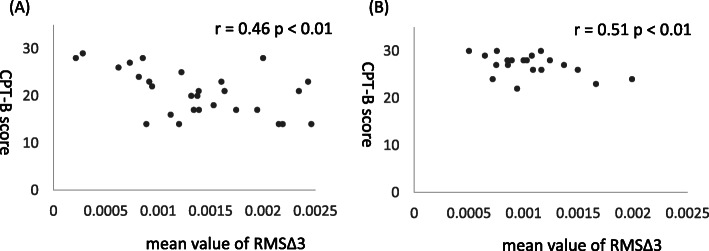
Correlations between the mean value of RMSΔ3 and the CPT-B in **a** older group and **b** young group. There were significant negative correlations between the RMSΔ 3 score and CPT-B score within both the older group and young group. RMSΔ3, root mean square of the third differentiation; CPT-B, The Card-Placing Test Part B

We found a significant negative correlation between the RMS∆ 3 score and CPT-B score within both the older group (*r* = − 0.46, *p* < 0.01) and the young group (*r* = − 0.51, *p* < 0.01) (Fig. 8).

## Discussion

In this study, we tested the hypothesis that some drivers with reduced spatial navigation ability cannot perform lane changes smoothly. The CTP-B score was significantly lower in the older group, which suggests that allocentric spatial perception is more difficult for older drivers, by adding processing in the allocentric reference frame evaluated by CPT-B to processing in the egocentric reference frame evaluated by CPT-A. Spatial navigation in driving tasks does not depend on a single frame, but requires the ability to flexibly switch and combine various spatial strategies according to the surrounding environment [28]. Our results replicate earlier studies that have demonstrated that attention focused on the self enhances perspective-taking [29, 30]. Studies targeting head trauma drivers have found that an impaired driving performance is accompanied by a declining frontal lobe function, self-recognition, and lack of awareness about their disease, which affects driving aptitude [31]. In the present study, the older group was able to maintain concentration on visual information input and driving force. In the self-centered spatial representation evaluated by CPT-A, it is necessary to integrate the projection position of the landmark on the retina and the information on the direction of the eyeball or neck trunk [32]. In other words, as processing in the egocentric reference frame is added to the process in the allocentric reference frame, older individuals must hold more information in working memory. Therefore, older is likely to be biased towards automatic information processing by the egocentric reference frame process, which suggests that it is difficult to smoothly switch between egocentric and allocentric reference frames. For healthy older people, an increase in self-centered space awareness whereby information processed using egocentric space perception strategies by the prefrontal cortex function would help to overcome this. No participants in the older group required more than 180 s to complete the TMT-B. However, this is indicative of deterioration of attention with age. Since there was no significant difference in other tests, we considered all participants to be capable of the driving necessary and sufficient for the DS task. There was no significant difference in motor function that does not need spatial navigation, and participants underwent a DS test.

The variance values of RMS Δ 3 and RMS Δ 3 were larger in the older group than in the young group. It has been reported that the entropy of the steering wheel operation when changing lanes at a constant vehicle speed is analyzed and the change of the speed in the lateral direction can be used as an index of driving skill [33]. Furthermore, the first derivative of speed and the second derivative of speed when doing clockwise curves at the timing of the driver within the statutory speed of the vehicle are related to the smoothness of driving and the level of skill [34]. Therefore, we can propose that the value of RMS ∆ 3 calculated from the change in the position in the lateral direction can be used as an indicator of the smoothness of the natural driving operation of the driver.

In this study, we assume that the operation is smoother as the sum of RMS ∆ 3 in each run is smaller. The older people had a large change in RMS ∆ 3 and a lower ability to smoothly control the vehicle. It has been reported that a driver predicts the future vehicle position based on the current vehicle information and operates while taking the distance from other vehicles into account [35]. When assuming that acquisition density decreases with age, and that older people can accurately memorize the arrangement of a certain object in the room, rotate the positional relationship of the object [36], driving skills are influenced by caution, reaction time, memory, cognitive function, mental state, visual function, disability of body function, and self-monitoring during driving. Among these factors, we think that self-monitoring is important when changing the lane. The driver’s perceptions of the positional relationship between one’s own and other vehicles predict the distance feeling to other vehicles and the position of the own vehicle after lane change [37]. For older people to perform these processes, they must access the working memory of the frontal lobe several times, use the spatial navigation ability to make a situation judgment and to drive, and monitor the trajectory at the time of lane change from an allocentric viewpoint. Therefore, when changing lanes, the evaluation value of RMS ∆ 3 of the older group and the variance value of RSM ∆ 3 are advantageously larger than those of young; although the cognitive function is within the normal range, it can be compensated consciously or unconsciously to secure safety. By adopting this strategy, the change in the smoothness of the lane change is significant. We propose that older people cannot cope with the speed change of the car body and the spatial navigation ability decrease.

Both the older group and the young group showed a significant negative correlation with RMS ∆ 3 and CPT-B scores. In a real-world driving situation, a driver must understand the space, be able to respond to changes in the surrounding environment, drive smoothly during acceleration and deceleration, and be able to perform smooth vehicle trajectory. From a functional point of view, the parietal lobe is involved in spatial navigation ability, as well as in visual-spatial processing [38]. Many studies have consistently reported that the reduction of executive function and attentional functions accompany general aging, and it increasingly longer to processing a sense of direction in the brain [7, 39, 40]. However, a decline in spatial navigation ability has also been associated with the posterior cingulate cortex and the retrosplenial cortex (RSC) [41–43]. In summary, despite the normal visual perception of the surrounding environment during driving in older people, they cannot predict the direction of travel and operate the vehicle properly using the cognitive process with the egocentric reference frame and the allocentric reference frame, so that they are in a state where stable driving conditions cannot be maintained. Atrophy of the prefrontal cortex means that vehicle operation cannot be performed properly, a stable operating condition cannot be maintained, and it is difficult to monitor one’s own vehicle [44]. A decrease in functional nodal betweenness was primarily located in the superior frontal lobe, right occipital lobe, and the global hubs [45]. In the older group, the egocentric frame and allocentric frame viewpoints cannot be switched, and the larger the change in the evaluation value of smoothness of vehicle movement during lane changing, the lower the CPT-B score. Although the results of this work cannot confirm what kind of smooth driving trajectories, simulator validity typically refers to the degree to which behavior in a simulator corresponds to behavior in real-world environments under the same conditions [46]; we presented the possibility of using DS to capture changes in spatial navigation ability and smooth driving trajectories. It is difficult to estimate the allocentric spatial navigation ability, and the possibility that the driving operations cannot be performed smoothly is considered as the allocentric reference frame process declines. In addition, such changes in driving skills are caused by a decline in spatial navigation capability of monitoring the movement of one’s own vehicle and appropriately switching while maintaining information processing for more than two types of information, as captured by the CPT-B results.

### Limitation

One of the limitations of this research is that it was not able to compare with actual car data. This means that we do not yet know if the smoothness of driving found in the present DS study applies to that of an actual car. Thus, we aim to conduct future experiments using actual cars to verify the present results. By doing so, there is a possibility that this will allow us to measure smooth driving trajectories with the actual car and thus overcome this limitation. Assessments of spatial navigation should be conducted using appropriate evaluation indices. Diversion to community-dwelling older, the purpose is to be able to participate in society safely in each region in a car society. It is expected that making the elderly driver aware of the spatial navigation ability from the physiological function test and the education and training stages will be a form of preventive safety to prevent traffic accidents caused by meandering driving. At the same time, as a function for cognitive and physical function support, it may be a necessary index for automakers to strengthen the accident prevention function for the elderly. It is expected that the creation of a common index for such a variety of occupations will facilitate cooperation. Furthermore, we want to extend the target to healthy people and use it as a support measure.

## Conclusions

We used a DS to investigate driving behavior and spatial navigation between healthy older people and healthy young people. We found a significant correlation in both groups between the smoothness of vehicle movement and spatial navigation, in the smoothness of vehicle movement between the young and old groups, and a significant difference in dispersion value. We can therefore suggest that driving smoothness is directly influenced by spatial navigation ability.

## Data Availability

The datasets during and/or analyzed during the current study available from the corresponding author on reasonable request.

## Abbreviations

BJLO: Benton Judgment of Line Orientation Test
CPT-A/B: The Card-Placing Test Part A and Part B
DS: Driving simulator
TMT-A/B: Trail Making Test Part A and Part B
RCPM: Raven’s Colored Progressive Matrices
RMS: Root mean square
